# Bridging the cultural divide: Simulated patient encounters to enhance medical students’ cross-cultural competences – A pilot study

**DOI:** 10.1371/journal.pone.0338266

**Published:** 2025-12-11

**Authors:** Aleksandra Walkowska, Piotr Przymuszała, Patrycja Marciniak-Stępak, Maria Nowosadko, Ewa Baum

**Affiliations:** 1 Centre for Foreign Language Tuition, Poznan University of Medical Sciences, Poznan, Poland; 2 Department of Medical Education, Poznan University of Medical Sciences, Poznan, Poland; 3 Department of Medical Simulation, Poznan University of Medical Sciences, Poznan, Poland; 4 Department of Social Sciences and the Humanities, Poznan University of Medical Sciences, Poznan, Poland; McGill University, CANADA

## Abstract

There is an increasing awareness of the need to include culture sensitive actions and knowledge in the medical curriculum to ensure quality care for the growing number of culturally and linguistically diverse patients in Polish healthcare system. However, the research on the availability and effectiveness of cultural training for Polish medical students still seems insufficient. This study was designed to evaluate the impact of a simulation-based learning intervention with simulated patients on medical students’ cross-cultural sensitivity, awareness and self-efficacy of their cross-cultural knowledge and skills as well as to collect participants’ opinions about the simulation-based learning experience. The pre-intervention and post-intervention questionnaires consisting of the validated Polish version of a 24-item Intercultural Sensitivity Scale and the self-developed questionnaires evaluating students’ self-efficacy of their abilities were used. Additionally, the post-intervention questionnaire was also used to collect general impressions from the training course. The questionnaires were answered by 70 students participating in a 15-hour cultural content module, including workshops and simulation sessions with simulated patients. Students’ intercultural sensitivity and self-efficacy regarding their cultural knowledge and skills improved significantly post-intervention. Students experienced the simulated encounters as authentic and useful in advancing their cultural understanding and cross-cultural communication skills. Coupled with appreciative students’ feedback, our analysis indicated that classes with simulated patients can serve as a reliable and effective learning method for enhancing cross-cultural competence of future doctors.

## Introduction

Teaching and evaluation of cultural competence in healthcare have generated much effort in the literature to develop tools and design models that would depict ideas and principles and provide practical strategies to develop culturally competent care [1–5]. Although there is a growing recognition of the need to provide cultural competence training to healthcare professionals, the construct itself seems difficult to define, and consequently, numerous concepts, such as cultural humility, cultural responsiveness, cultural effectiveness, intercultural sensitivity, etc., have been in use [6]. The term cultural competence was first introduced into healthcare by Cross et al. [7] who saw it as a set of congruent behaviours, attitudes and policies that enabled professionals to act effectively in cross-cultural interactions. Tervalon and Murray-Garcia [8] favour the term cultural humility over a traditional concept of cultural competence and emphasise the ongoing process of acquiring cultural knowledge and understanding during respectful interactions with culturally diverse patients. Dreachslin et al. [9] stress the significance of cultural awareness and cultural sensitivity as the crucial steps to developing culturally competent healthcare. The first term relates to the recognition of distinctive cultural traits and needs of individuals, including acknowledgement of own biases and prejudices; the second one pertains to the empathetic response towards those needs. Then, Chen and Starosta [10] suggest the notion intercultural communication competence as an umbrella concept for the cognitive, affective and behavioural ability of an individual to engage in intercultural communication. Intercultural sensitivity, which constitutes the affective aspect of intercultural communication competence, seems to be the crucial element of the competence and enables individuals to comprehend how other people may differ in the way they behave, perceive the world and express emotions. Intercultural sensitivity has been defined as the ability of an individual to positively display understanding and appreciation towards perceived differences, which is further specified in six elements: self-esteem, self-monitoring, open-mindedness, empathy, interaction involvement and non-judgement [10]. As observed by Campinha-Bacote [5], obtaining cultural competence is a life-long education process. In countries such as Poland, which, as remarked by Pollock [11], has taken impressive steps to re-build a multicultural and tolerant society following the collapse of a ‘*monocultural totalitarian regime’* [11], the role of education to re-engineer the atmosphere of tolerance and inclusion has been particularly significant. In a similar vein, Antczak [12] stresses proper education process as a major path towards inclusion of immigrants into local communities.

The complexity of concepts and theories, however, as well as the complications in articulating one single definition of cultural competence in healthcare contribute to difficulties in integrating cross-cultural training into the medical curricula and selecting optimal teaching approaches [13]. In fact, methods of instruction to enhance cultural competence vary and healthcare faculties, understanding the increasing significance of the cultural aspect of medical training, have been using a wide variety of learning modalities and activities to equip students with cultural competence. These include, for example, lectures, tutorials, reading literature from cultural journals, case studies, class debates, class discussions, discussions with guest lecturers, group presentations, storytelling, videos, quizzes, role-plays, clinical experience, students exchange programmes, cultural immersions with local and international communities as well as simulation methods [14–16].

One of the simulation-based learning methods to facilitate teaching and evaluating the skills and the quality of students’ performances are simulations with human actors, known as simulated patients (SPs). Seen by Barrows [17] as individuals who have been meticulously trained to portray a real patient, they are believed to bring numerous advantages and possibilities for medical students and educators. Firstly, similarly to other simulation-based methods, they allow for the adoption of a student-directed model of education with a considerable increase in the engagement of a learner. Although simulation methods will never fully replace the work with real patients in this respect [18], this fully interactive and immersive technique offers a safe, non-threatening and controlled environment for medical students to increase their self-efficacy and develop competences [19–23]. Next, the mistreatment of a patient or the embarrassment of a learner, both resulting from the latter’s insufficient knowledge and/or confidence, is minimised during a simulated encounter with an actor, whereas the ability to manage stress and prepare students emotionally to deal with real-life clinical situations is markedly increased [17,24]. Then, constructive feedback provided by patients in the form of post-simulation debriefing sessions also strengthens the learning objectives by helping students to understand the encounter through discussion and reflection [20,22–25]. The availability of SPs in terms of time and location seems unhindered, which eases the class arrangement; their performances are highly standardisable, which allows to achieve a very congruous portrayal of the problem and, what is very important, the scenarios they present can be re-enacted a number of times, helping to solidify the learning and/or assessment objectives [17,23,26]. Then, by incorporating appropriate content into the scenarios, teachers can decide on the level of difficulty of the training session by adding or removing complications in the patient’s condition to achieve the intended learning outcomes [17]. Moreover, SPs can be trained to reproduce any specific human traits, such as age, race, educational level, psychological, emotional and physical condition, etc. [27]. The realism of the encounter can be enhanced through the introduction of specific sociocultural elements into the scenario, such as culturally specific names, language, non-verbal communication styles, mannerisms, clothes, etc., which seems particularly relevant in designing cultural content scenarios [28]. Finally, the sessions with SPs remain highly valued by medical students for the authenticity of the encounters and, most of all, for the immersive model of training they offer, which allows students to apply theoretical knowledge into practice during real-life interactions with patients [15,20,29–31]. Consequently, the desire for more educational interventions with SPs has been expressed by students, who view them as an opportunity to increase the self-efficacy of their communication skills and a chance to experience emotions, which should help them deal with stress in their future real-life clinical encounters [21,24].

Nevertheless, certain barriers to simulation programmes with actors have also been noticed. It has been argued, for example, that a good quality session necessitates a long period of training delivered to patients. This opinion, however, has been challenged by Barrows [17], who claims that by focusing on the understanding of emotional aspects of the case rather than memorising the role as a script, the training time may be limited to even up to three hours [17]. Other presumed disadvantages of simulation-based learning are its financial and human resources, including actors’ fees and training as well as staff involvement necessary to design and foster scenarios, develop evaluation tools and coordinate the entire simulation programme [23,32]. Finally, actors are assumed to be able to demonstrate a limited scope of physical symptoms. On the other hand, it has been argued that the “*range of findings that can be simulated on physical examination is probably greater than most faculty realize”* [17].

To conclude, although sessions with SPs are never expected to replace clinical encounters with real patients, they are believed to enhance the educational effects by letting students experience contact with specific patients and their problems rather than hoping that students will eventually encounter them in a clinical setting [17,33]. Additionally, due to their capacity to generate human as well as environmental fidelity by introducing culture-specific elements to the scenarios, simulated interventions seem to be a valuable learning modality to teach future physicians how to provide culturally sensitive care [28]. Numerous studies demonstrate how simulations with actors contribute to the increase in students’ cultural awareness and sensitivity, help them to develop their culture-sensitive communication skills, boost their self-confidence and show them how to adapt their actions to their patients’ diverse cultural behaviours [28]. Most importantly, however, the literature emphasises noticeably high satisfaction with this learning method among students, who value the culturally tailored health interventions with actors for their authenticity and usefulness [28].

Despite still relatively high demographic homogeneity of Poland, the last decades have brought a visible diversification of the Polish population. A growing number of patients with different cultural backgrounds have become part of the Polish healthcare system and the need to equip medical students with the ability to comprehend and apply culture-sensitive knowledge and actions to provide culturally congruent care has become evident. Consequently, numerous studies have focused on medical cross-cultural training of Polish healthcare professionals [34–45]. Nevertheless, there still seems to be a shortage of research on the effectiveness of training methods, including sessions with SPs, in enhancing the cross-cultural competence of Polish medical students. To the authors’ best knowledge, only one Polish study focused on the assessment of the results of cross-communication training on the intercultural competence of Polish healthcare students [41]. In their paper, Majda et al. [41] presented the effects of an intercultural workshop on nursing students’ knowledge, skills and attitudes, emphasising the need for intensification of cross-cultural training programmes for future nurses as well as integrating a greater number of active teaching strategies. There seems to be no research, however, evaluating the effectiveness of simulation-based learning interventions on cross-cultural competences of future Polish physicians. Therefore, this study’s objective was to design a cultural content training with SPs to evaluate its impact on medical students’ intercultural sensitivity and their self-efficacy of their cross-cultural knowledge and skills. The second aim was to collect students’ impressions of the encounters, including atmosphere, authenticity, attractiveness, involvement of students and teachers, quality of acting demonstrated by the simulated patients and their suggestions for potential improvement of the classes.

## Materials and methods

### Study design

“*A cross-cultural patient and how to communicate with them*” class was introduced in the academic year 2024/2025 as an elective course for students of medical faculty at Poznan University of Medical Sciences. The elective module was available for students ranging from years 2–5. A 15-hour course consisted of two parts, with the first unit including a 5-hour introductory workshop devoted to the issues of migration, cultural diversity and sensitivity. During this session, participants engaged in discussions, talked about identity, stereotypes and cultural barriers, watched video demonstrations, engaged in role-plays, etc. In the second part of the course, which was held in the Medical Simulation Centre as two separate sessions, the students were familiarized with the LEARN protocol [46] as a valuable tool when communicating with a cross-cultural patient as well as with best practices while collaborating with interpreters during medical encounters [47] before being presented with five cultural content scenarios.

The first scenario involved the case of a Ukrainian woman fleeing from her home in Odessa following the Russian invasion of Ukraine. Although having found safety and protection for herself and her two young children in Poland, the woman suffers from psychological distress. The student was in the role of a family doctor, and their task was to recognize the mental health impacts of war and forced migration and offer support and help.

In the second scenario, students met a pregnant Muslim woman, who additionally did not speak Polish, wanting to consult with her doctor about the safety of fasting during the coming month of Ramadan. The task for the student taking the role of a doctor was to inform the patient, whose blood tests revealed a slightly increased blood sugar level, about the risks of fasting, showing respect and understanding for her spiritual needs at the same time.

The third scenario contained the case of a patient from Thailand, who also did not speak Polish, complaining of lower back pain resulting from a strenuous gym session. Additionally, the patient, who is a young student away from home and family, demonstrates some mild signs of adaptation problem behaviours, such as homesickness, loss of support system, culture shock, etc. The doctor’s task was to identify those problems and offer support and help.

The fourth scenario focused on the Jehovah’s Witness (JW) parent who refused a blood transfusion for his 7-year-old son, a road accident victim. The student’s role (as a doctor in the scenario) was, at first, to attempt to obtain the father’s consent for transfusion**,** provide, in a supportive and empathetic manner, comprehensive information about possible consequences of lack thereof, and ultimately, in view of the father’s definite refusal planned in the scenario**,** refer to applicable legal regulations in order to save the child’s life.

The fifth scenario involved a case of a patient, a tourist holidaying in Poland, who needs medical attention following an insect bite and a resulting mild allergic reaction. As the patient speaks no Polish or English, her relative, who is a fluent Polish speaker, offers to help as an interpreter. The doctor’s task was to interview the patient and provide support and treatment with the assistance of an interpreter.

The majority of SPs participating in the scenarios were members of a professional team of SPs cooperating with the Medical Simulation Centre of our university on a permanent basis. Having been periodically engaged in numerous trainings to help them accurately portray symptoms, behaviour and history as well as construct valuable feedback, the university SP team is well prepared to consistently represent the same roles in a realistic and repeatable way for a standardised experience. Moreover, to maintain standardization level between sessions during the course, each individual scenario was consistently performed by the same SP.

Additionally, to increase the credibility and attractiveness of the scenarios, the patient in the third scenario was a second-year medical student from Thailand enrolled in the English programme at our university, who also provided valuable cultural insight into the scenario and was carefully trained and instructed by the first author on how to perform the role of a SP and deliver feedback to students during a debriefing session. The cultural content of the second scenario was also reviewed by a member of a local Muslim community in Poznan, who provided consultation on clothing, non-verbal aspects of communication, rules pertaining to gender relations as well as relevant religious and spiritual issues.

Each scenario involved one active student participant, taking the role of a doctor, and two other students, who were also present in the consultation room, but their role was reduced to observing verbal and non-verbal communication patterns. Due to limited amount of time dedicated to the classes, we were not able to provide all students with the opportunity to become actively involved in a scenario. However, the rest of the students were able to watch the encounter on the screen in the debriefing room, where the debriefing session based on the Pendleton model [48] was conducted after each scenario. To evaluate the experience, a mixed study design with pre-test/post-test assessment was utilized and all medical students who participated in the course were presented with the pre-test and post-test surveys. Before starting this study, verbal informed consent for participation was collected from the students, who were also informed about the study aims. They were notified by the tutors that their participation in the study was completely voluntary and that by completing and returning the surveys they agreed to participate in it.

### Research tools

**Fig 1:**
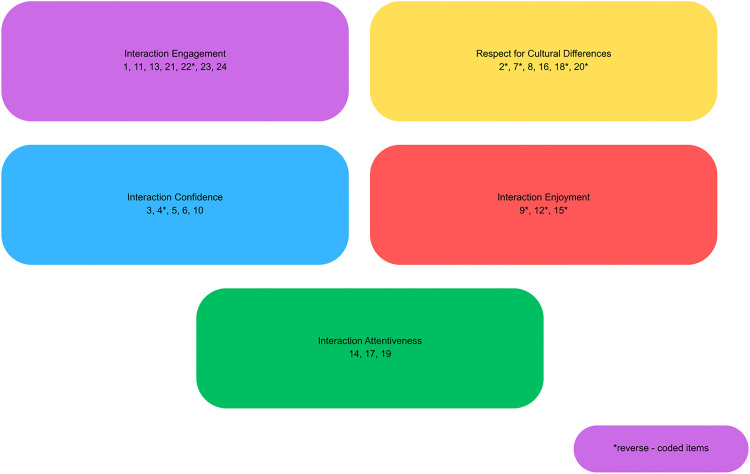
Visualisation of the Polish adaptation of the Intercultural Sensitivity Scale (ISS).

The pre-intervention and post-intervention questionnaires utilized the validated Polish version of a 24-item Intercultural Sensitivity Scale (ISS) to investigate students’ intercultural sensitivity before and after the training [49]. The Polish adaptation, like the original English version, is a five-point Likert-type instrument (5 = strongly agree, 4 = agree, 3 = uncertain, 2 = disagree, 1 = strongly disagree) comprising five sub-scales: Interaction Engagement (items 1, 11, 13, 21, 22, 23 and 24), Respect for Cultural Differences (2, 7, 8, 16, 18 and 20), Interaction Confidence (items 3, 4, 5, 6 and 10), Interaction Enjoyment (items 9, 12 and 15) and Interaction Attentiveness (14, 17 and 19). The visualisation of the ISS is illustrated in Fig 1. Additionally, both pre- and post- intervention surveys contained questions asking students to rate their self-assessed cross-cultural competencies formulated according to the learning outcomes intended to be achieved by medical students according to the Ordinance of the Ministry of Science and Higher Education [50]. Questions were rated on a five-point Likert scale ranging from 1 (very poor) to 5 (very good). The third section of the post-intervention survey, containing both closed and open questions, attempted to collect students’ general impressions of the training course, their opinions on particular aspects of the course as well as their perception of the effectiveness of the simulations. The surveys were prepared by the first author and reviewed by the other authors, who have considerable expertise in medical education and communication training. They were also pilot-tested in terms of clarity on a sample of 10 medical students who were not participating in the study. Both questionnaires were anonymous, and the students were also asked to disclose their study year, age and gender. Data were collected between November 2024 and January 2025.

### Data analysis

Data obtained from the questionnaires were subsequently transcribed and analysed depending on their character. The qualitative data obtained during the course of the study were content analysed by two independent researchers. The quantitative were statistically analysed using the PQStat and Statistica software packages with the use of the following tests: the Wilcoxon signed-rank test, the Mann-Whitney U test and Spearman’s correlation coefficient, as applicable.

### Ethical considerations

The protocol of this study was presented to the Bioethics Committee of the Poznan University of Medical Sciences, which confirmed that their approval was not legally required for the present research to be conducted (Decision No. KB – 915/22). Before engaging in the study, students were also informed about its objectives and the scientific character as well as their voluntary participation in it.

## Results

### Demographic characteristics of the participants of the study

Out of 71 participants of the classes, only one student did not return the post-intervention questionnaire and was, therefore, excluded from the study. The age of the participants ranged from 19 to 28 (mean 20.71 ± 1.72). Among the 70 students who completed both surveys, 49 (70%) were female and 21 (30%) were male. The detailed demographic characteristic of the group of students participating in the study is presented in Table 1. It should also be noted that due to the elective character of the course, the authors had no influence on who enrolled in the course and, thus, participants’ age, study year and gender.

**Table 1:** Demographic characteristics of students (n = 70).

		n	%
Gender	Male	21	30
	Female	49	70
Study year	Year 2	62	88.6
	Year 3	2	2.9
	Year 4	1	1.4
	Year 5	5	7.1

### Intercultural Sensitivity Scale ratings before and after the course

As illustrated in Table 2, the pre-post analysis of the ISS revealed a statistically significant increase in students’ intercultural sensitivity level as measured by the total ISS scale score (p < 0.033). Moreover, further analysis of the ISS domains showed changes in cultural sensitivity scores on the individual subscales but only in the Interaction Attentiveness subscale was the difference statistically significant (p < 0.001). The Interaction Attentiveness score after the course was also positively correlated with the age of participants (p = 0.011, *r* = 0.29). There were no statistical differences in this aspect before the course. Additionally, although the age of the students was positively correlated (*p* = 0.046, *r* = 0.23) with their score on the Interaction Engagement subscale before the intervention, it was no longer observed after the course. The score of female students in the Interaction Engagement domain was also higher in the post-intervention test than the results of male respondents (p = 0.019). No other differences between pre and post-intervention scores in terms of participants’ gender, age and year of study were noted.

**Table 2:** Comparison of ISS total and ISS domains before and after the course.

Intercultural Sensitivity Scale ISS		n	Mean ± SD	Me (Q1; Q3)	p-value*
**Interaction Engagement**	PRE	70	26.99 ± 3.36	27 (25; 28.5)	0.141
	POST	70	27.19 ± 3.94	27 (26; 29)	
**Respect for Cultural Differences**	PRE	70	25.73 ± 3.31	26 (24; 29)	0.405
	POST	70	24.94 ± 4.47	26 (22.5; 28)	
**Interaction Confidence**	PRE	70	16.14 ± 3.25	16 (14; 18)	0.097
	POST	70	16.84 ± 3.28	17 (14; 20)	
**Interaction Enjoyment**	PRE	70	11.69 ± 1.82	12 (11; 13)	0.734
	POST	70	11.69 ± 2.03	12 (11; 13)	
**Interaction Attentiveness**	PRE	70	11.04 ± 1.96	11 (10; 12)	<0.001
	POST	70	11.96 ± 2.12	12 (11; 13)	
**Total**	PRE	70	91.59 ± 7.61	91 (86; 98)	<0.033
	POST	70	92.62 ± 7.22	94 (86;101.75)	

*p-values significant at level of 0.05; Me – median, Q1 – lower quartile; Q3 – upper quartile.

### Students’ self-efficacy ratings of their cross-cultural knowledge and skills before and after the course

Students’ pre- and post-intervention self-efficacy ratings are presented in Table 3. As shown, after the course, respondents assessed their abilities to apply relevant culture’s caring actions and knowledge significantly higher in regard to all evaluated learning outcomes. Some differences in students’ responses were also identified based on their demographics.

**Table 3:** Comparison of students’ self-efficacy ratings before and after the intervention.

Students’ self- efficacy ratings before and after the course (1 = very poor; 5 = very good)		n	Mean ± SD	Me (Q1; Q3)	p- value
Knowledge and understanding of the impact of social disparities and cultural differences on health	PRE	70	3.54 ± 0.85	3 (3; 4)	<0.001
	POST	70	3.90 ± 0.80	4 (3; 4)	
Knowledge and understanding of socio- cultural barriers	PRE	70	3.53 ± 0.72	4 (3; 4)	<0.001
	POST	70	3.99 ± 0.65	4 (4; 4)	
Knowledge and understanding of communication methods for building trusting relationship with patients and their family	PRE	70	3.33 ± 0.86	3 (3; 4)	<0.001
	POST	70	4.06 ± 0.63	4 (4; 4)	
Knowledge and understanding of the importance of verbal and non-verbal communication	PRE	70	3.77 ± 0.87	4 (3; 4)	0.001
	POST	70	4.21 ± 0.66	4 (4; 5)	
Knowledge and understanding of cultural, ethnic and national variables	PRE	70	3.13 ± 0.88	3 (3; 4)	<0.001
	POST	70	3.79 ± 0.72	4 (3; 4)	
Identifying subjective socio-cultural needs of patients	PRE	70	3.09 ± 0.91	3 (2.25; 4)	<0.001
	POST	70	3.76 ± 0.71	4 (3; 4)	
Building atmosphere of trust	PRE	70	3.70 ± 0.79	4 (3; 4)	<0.001
	POST	70	4.19 ± 0.73	4 (4; 5)	
Talking to patients and their family with the use of active listening techniques and empathizing	PRE	70	3.64 ± 0.87	4 (3; 4)	0.001
	POST	70	4.01 ± 0.81	4 (4; 5)	
Obtaining informed consent from patients	PRE	70	3.49 ± 0.81	4 (3; 4)	<0.001
	POST	70	4.05 ± 0.92	4 (4; 4)	
Communicating with patients in English (B2+ on the CEFR scale)	PRE	70	3.61 ± 1.04	4 (3; 4)	<0.001
	POST	70	4.09 ± 0.88	4 (3.25; 5)	
Knowledge of forms of interactions in speech	PRE	70	4.17 ± 0.76	4 (4; 5)	0.007
	POST	70	4.43 ± 0.60	4 (4; 5)	
Identifying culture-based communication patterns	PRE	70	2.81 ± 0.84	3 (2; 3)	<0.001
	POST	70	3.64 ± 0.76	4 (3; 4)	
Using language understandable for patients from distant cultural backgrounds	PRE	70	3.09 ± 0.83	3 (2.25; 4)	<0.001
	POST	70	3.74 ± 0.81	4 (3; 4)	
Talking to patients with the assistance of an interpreter	PRE	70	2.94 ± 1.18	3 (2; 4)	<0.001
	POST	70	3.94 ± 0.70	4 (4; 4)	
Identifying needs of patients suffering from war-related trauma	PRE	70	2.07 ± 0.82	2 (2; 2.75)	<0.001
	POST	70	3.17 ± 1.04	3 (2; 4)	
Recognizing and including in the treatment socio-cultural needs and expectations of patients	PRE	69	2.91 ± 0.80	3 (3; 3)	<0.001
	POST	69	3.71 ± 0.70	4 (3; 4)	
Managing patients’ refusal of recommended treatment due to their beliefs	PRE	70	2.49 ± 0.88	2 (2; 3)	<0.001
	POST	70	3.27 ± 1.01	3 (3; 4)	
Managing patients’ refusal of their child’s recommended treatment due to their beliefs	PRE	70	2.10 ± 0.89	2 (1; 3)	<0.001
	POST	70	3.23 ± 1.18	3 (2; 4)	
Talking to patients attached to natural treatment methods and traditional health beliefs	PRE	70	2.33 ± 0.99	2 (2; 3)	<0.001
	POST	70	3.23 ± 0.92	3 (3; 4)	
Readiness to deliver care to cross-cultural patients	PRE	69	2.94 ± 0.90	3 (2; 3.75)	<0.001
	POST	69	3.81 ± 0.79	4 (3; 4)	

Before the course, female students ranked their abilities to identify culture-based communication patterns higher than their male colleagues (*p* = 0.011), and that was the only statistically significant gender difference observed before the intervention. After the course, female students gave significantly higher ratings to their knowledge and understanding of communication methods for building a trusting relationship with patients and their family (*p* = 0.033), their knowledge and understanding of the importance of verbal and non-verbal communication (*p* < 0.001) as well as their capabilities to talk to patients with the assistance of an interpreter (*p* = 0.039). Male students, on the other hand, rated themselves significantly higher than females in communicating with patients in English (*p* = 0.005), managing patients’ refusal of recommended treatment due to their beliefs (*p* = 0.002) and managing patients’ refusal of their child’s recommended treatment due to their beliefs (*p* = 0.002).

Students’ year of study was positively correlated with the pre-intervention rankings of their knowledge and understanding of socio-cultural barriers (*p* = 0.041, r = 0.24), knowledge and understanding of communication methods for building a trusting relationship with patients and their family (*p* < 0.001, r = 0.41), knowledge and understanding of the importance of verbal and non-verbal communication (*p* < 0.001, r = 0.42), identifying subjective socio-cultural needs of patients (*p* = 0.002, r = 0.35), building atmosphere of trust (*p* = 0.001, r = 0.38), managing patients’ refusal of their child’s recommended treatment due to their beliefs (*p* = 0.035, r = 0.25) and talking to patients and their family with the use of active listening techniques and empathizing (*p* = 0.020, r = 0.27). After the intervention, a positive correlation with students’ study year was found only in the case of talking to patients attached to natural treatment methods and traditional health beliefs (*p* = 0.048, r = 0.23).

There was also a positive correlation between the age of respondents and their pre-intervention assessment of their knowledge and understanding of communication methods for building a trusting relationship with patients and their family (*p* = 0.021, r = 0.27), knowledge and understanding of the importance of verbal and non-verbal communication (*p* = 0.003, r = 0.34), identifying subjective socio-cultural needs of patients (*p* = 0.011, r = 0.30), building atmosphere of trust (*p* = 0.024, r = 0.26) and managing patients’ refusal of their child’s recommended treatment due to their beliefs (*p* = 0.032, r = 0.25). The only difference in this aspect observed after the course was the positive correlation of the age of students with their knowledge and understanding of cultural, ethnic and national variables (*p* = 0.043, r = 0.24).

### Students’ opinions about the course

The course and its individual aspects received very high ratings from participants, who, in the majority, assessed them as good and very good (Table 4). Students valued the intervention for its usefulness, enjoyed the atmosphere during the course and appreciated the feedback received after each scenario and the possibility of observing their fellow students. They were impressed by the way SPs portrayed their roles and, in fact, the performance of SPs received the highest rating among all the aspects. The lowest rating, on the other hand, was given to students’ own engagement during the class; however, it was still assessed as good or very good by over 82% of participants. In terms of gender, female students significantly more positively valued scenarios used during the course (*p* = 0.008) and the usefulness of the classes in enhancing cross-cultural competence (*p* = 0.010) compared to male students. The age of participants was positively correlated with their evaluations of the course usefulness (*p* = 0.003, r = 0.34), scenarios used during the classes (*p* = 0.047, r = 0.23), possibility of observing other students (*p* = 0.041, r = 0.24) and feedback from other students (*p* = 0.012, r = 0.29). No differences in terms of participants’ study year were observed in their evaluation of the particular aspects of the course.

**Table 4:** Students’ assessment of individual aspect of the course.

Ratings of the Individual Course Aspects	n	Mean ± SD	Me (Q1; Q3)	Good and Very Good
General impression from the course	70	4.81 ± 0.42	5 (5;5)	98.57%
Usefulness of the course in enhancing cross-cultural competence	70	4.80 ± 0.46	5 (5; 5)	97.14%
Course atmosphere	70	4.78 ± 0.56	5 (5; 5)	95.71%
Teachers conducting the class	70	4.90 ± 0.38	5 (5; 5)	97.14%
The way SPs presented their roles	70	4.91 ± 0.28	5 (5; 5)	100%
Layout and equipment of the consultation room	70	4.70 ± 0.52	5 (4; 5)	97.14%
Scenarios used during the course	70	4.75 ± 0.46	5 (5; 5)	98.57%
Possibility of observing other students	70	4.74 ± 0.52	5 (5; 5)	95.71%
Feedback from teachers	70	4.80 ± 0.49	5 (5; 5)	95.71%
Feedback from SPs	70	4.85 ± 0.39	5 (5; 5)	98.57%
Feedback from other students	70	4.61 ± 0.74	5 (4.25; 5)	90.00%
Own engagement during the class	70	4.24 ± 0.80	4 (4; 5)	82.85%
Other students’ engagement during the class	70	4.42 ± 0.75	5 (4; 5)	90.00%

Students’ level of agreement with statements directly describing the course, presented in Table 5, also showed a very positive reception of the intervention. In fact, all students participating in the training agreed or definitely agreed with the comment that the classes with SPs are a good idea and should be organized more often. Similarly, all participants were impressed with the character credibility achieved by SPs. Furthermore, a vast majority of them agreed that the course, which constituted an engaging experience and was conducted in a learning-stimulating atmosphere, helped them to improve their cross-cultural competencies.

**Table 5:** Students’ level of agreement with the statements characterizing the course.

Statements Describing the Course (1 = Definitely Disagree; 5 = Definitely Agree)	n	Mean (SD)	M (Q1; Q3)	Agree or Definitely Agree
Classes with simulated patients are a good idea and should be organized more often.	70	4.88 ± 0.32	5 (5; 5)	100%
My cross-cultural competencies improved after the course with simulated patients.	70	4.68 ± 0.49	5 (4; 5)	98.57%
Knowledge and skills from the course will be useful in my future professional career.	70	4.74 ± 0.50	5 (5; 5)	97.14%
After the course, it will be easier for me to talk with real patients.	70	4.60 ± 0.57	5 (4; 5)	95.71%
Atmosphere during the class stimulated learning process.	70	4.84 ± 0.40	5 (5; 5)	98.57%
Simulated patients were well prepared and credible while playing their roles.	70	4.85 ± 0.35	5 (5; 5)	100%
I felt like talking to real patients.	70	4.70 ± 0.62	5 (5; 5)	94.28%
The course was an engaging experience for me.	70	4.81 ± 0.49	5 (5; 5)	98.57%
Scenarios were not demanding enough and did not constitute any challenge for me.	70	2.01 ± 1.13	2 (1; 3)	14.28%
Scenarios involved situations I might come across in future job.	70	4.64 ± 0.53	5 (4; 5)	97.14%
Talking to simulated patients, I felt like a real doctor.	70	4.01 ± 0.84	4 (4; 5)	75.71%
During the course, I felt motivated to do my best to help patients.	70	4.50 ± 0.65	5 (4; 5)	94.28%
Possibility of observing other students created an additional occasion to learn.	70	4.61 ± 0.76	5 (4; 5)	92.85%
Possibility of observing other students gave me a chance to notice my own mistakes.	70	4.48 ± 0.75	5 (4; 5)	90.00%
Presence of other students as observers was not a problem for me.	70	4.02 ± 1.15	4 (3; 5)	71.42%
Presence of other students was intimidating and distracting.	70	2.30 ± 1.26	2 (1; 3)	18.57%
I believe I gained a lot from participating in the course.	70	4.72 ± 0.53	5 (5; 5)	95.71%
Feedback from simulated patients and other students made me realize things I did not notice before.	70	4.62 ± 0.61	5 (4; 5)	92.85%
I would willingly participate in similar classes in the future.	70	4.62 ± 0.62	5 (5; 5)	95.71%

Statistical analysis revealed some differences in the agreement level regarding students’ gender, age and study year. Firstly, females significantly stronger than their male friends believed that after the course, it would be easier for them to talk with real patients (*p* = 0.012) and that scenarios involved situations they might come across in their future jobs (*p* = 0.015). Secondly, a positive correlation was noticed between the age of respondents and the belief that the possibility of observing other students created an additional occasion to learn (*p* = 0.016, r = 0.28) and that was the only age-related difference observed. Finally, most statistical differences were identified with regard to respondents’ study year. There was a positive correlation between the year of study of participants and their level of agreement with the statement that the possibility of observing other students gave them a chance to notice their own mistakes (*p* = 0.019, r = 0.27) and that during the course, they felt motivated to do their best to help patients (*p* = 0.010, r = 0.30). On the other hand, students’ study year also positively correlated with their opinion that scenarios were not demanding enough and did not constitute any challenge for them (*p* = 0.013, r = 0.29). However, it should be noted that only 14.28% of respondents agreed or definitely agreed with this opinion concerning the scenarios.

Lastly, in the final part of the questionnaire, students were asked to reflect on their impressions of the course. Their answers to the open questions in the survey showed that participants noticed an array of advantages of the intervention. Most importantly, they valued the possibility of practicing communication through active engagement in a real-life situation with a patient without worrying about making mistakes. This was especially appreciated by second-year students who had generally limited previous experience with simulated scenarios. For many students, the class was also an opportunity to practice communication in English and was indicated as a great tactic to overcome a language barrier. Moreover, students valued the class for its culture-based content and admitted it helped them understand and learn a lot about different beliefs, values, and lifeways. Respondents appreciated the diversity of the scenarios, the variety of engaging problems they depicted and the realistic portrayals delivered by SPs, who were described as dedicated professionals with outstanding acting skills. A great appreciation was shown towards the feedback after the scenarios, which was described as helpful and informative. Students were grateful for the opinions and reflections expressed by teachers and SPs, but many also pointed to the great significance of feedback from students of higher years of study, who willingly shared their experiences during the class. Finally, students enjoyed the atmosphere during the course, which was characterized as motivating and inspiring.

*“It was my first ever experience when I actually talked to a patient and I could talk to her without worrying I could say something wrong that may hurt her.”* (F/20)

*“It helped me understand that no matter what your nationality or religion, you need to remain professional in every situation.”* (F/20)

*“Feedback was very useful and was conducted in a very delicate and tactful manner and it helped me understand some emotions and behaviors.”* (F/19)

Although the majority of students noticed no drawbacks of the course, a few shortcomings were mentioned. Among the things students did not particularly like about the classes was the fact that they were scheduled for late afternoons and the individual sessions were quite long. Some participants felt the first-day workshop and after-scenario feedback sessions were also too lengthy as this time could have been accommodated to fit more scenarios, which would have allowed more students to participate as doctors. The fact that only a small number of them could try themselves in a physician’s role was also indicated as a disadvantage. Some students also admitted that the perspective of knowing that others were watching them was a significant source of stress. However, as some remarked, the stress was mostly present in the beginning and subsided once in the consultation room. Finally, several participants reflected that some of the scenarios were too emotional for them.

*“Some scenarios were so soul-stirring. It was hard to stay calm during the Jehovah’s Witness scenario.”*(F/20)

*“The Ukrainian patient made me realize that there are so many people who experience such problems and I feel helpless because we are not able to help them all.”* (M/20)

*“The classes were too long, but this disadvantage was outweighed by the great atmosphere of the meetings.”* (F/21)

Finally, students were asked to indicate changes that could be introduced to make the course even better. Firstly, a strong demand was made to increase the number of opportunities during medical studies that would prepare future doctors to communicate with patients from distant cultural backgrounds. Students stressed that the greater amount of classes would make them feel more confident in their future jobs as physicians and bring many profits to patients. They also complained about the elective character of the course and believed it should be obligatory for every medical student. Finally, they saw the sessions with SPs as a great opportunity to practice their language skills and suggested more scenarios in which they would need to use and practice English.

*“I learned a lot about different cultures but not enough – they should make more classes of this kind.”* (F/22)

*“The course should be included in the syllabus instead of being offered only as an elective as we’re having more and more patients from different cultures.”* (M/23)

## Discussion

With globalization, Poland is experiencing more and more diversity in its population structure, which calls for promoting culturally inclusive environments in many aspects of social life, including healthcare. Medical schools are, therefore, expected to search for the most effective tools to teach cross-cultural healthcare that would equip students with the knowledge and skills essential to provide care to patients from distant cultural backgrounds. This study aimed to assess the impact of the classes with SPs on medical students’ cultural sensitivity and self-efficacy of their cross-cultural knowledge and skills and participants’ views and opinions about the course.

Findings from this paper indicate the already relatively high level of pre-test cultural sensitivity among medical students included in the study, which seems consistent with other studies employing the questionnaire to evaluate the cross-cultural sensitivity of healthcare professionals [51–54]. The high baseline level of cultural sensitivity of our participants may be linked to the voluntary character of the course, which might have mostly attracted students interested in finding out how to interact with patients from distant cultures. Moreover, although a similar study conducted by Unver et al. [55] indicated no significant differences between cultural sensitivity scores before and after simulations, our results showed participants’ statistically significant improvement in the post-test total score and a statistically significant increase in the Interaction Attentiveness scale. For instance, Liu et al. [56] observed that attentiveness, which is the ability to receive and respond to messages properly during intercultural interaction, is strongly affected by students’ engagement and confidence in intercultural communication. In view of this opinion, the accompanying slight rise in the scores on the interaction engagement and interaction confidence subscales of our survey seems expected. Surprisingly, however, our findings show a little decline in participants’ scores on the Respect for Cultural Differences subscale, which, however, was not statistically significant. Portalla and Chen [57] remark that respect in intercultural communication should be seen as the outcome of admiration and approval and it could also be viewed as the ability of an individual to put another person’s interests first. The slight drop on this subscale may be considered in relation to students’ responses in the open questions section of the survey, where they referred to emotional distress they had experienced after the scenario with JW’s father, who refused a life-saving treatment of his child. Even though participants showed their understanding of JWs’ right to refuse blood on religious grounds, they expressed little approval of such a decision in a life-threatening situation concerning a child and expressed resentment toward JWs’ viewpoint. A recent study by Domaradzki et al. [37] similarly indicates that future nurses and midwives who, although being aware of the legal aspects of providing care to JW patients, show limited approval of JWs’ stance on blood transfusion refusal.

Next, following the intervention, students’ self-efficacy of their cross-cultural knowledge and skills considerably increased in all assessed learning outcomes. Research has demonstrated the multifaceted factors that affect learners’ self-efficacy and has indicated an interplay of key predictors. For instance, Bandura [58,59] evidences four main self-efficacy sources. Firstly, he states that one of the most effective ways to build a strong sense of efficacy is through performance accomplishments and repeated success. Furthermore, repeated failures can undermine or weaken self-efficacy; occasional failures, on the other hand, can strengthen it [59]. The simulated encounters in our study allowed students to immerse themselves in real-life situations in which they could master their skills of communicating with patients. Not without significance is the fact that most of the participants had limited previous experience of patient-doctor interactions, and for many, talking to a patient from a different culture was a highly unfamiliar encounter. The occasional failures they experienced as a consequence, however, could have been perceived as a chance to persist through challenges, build resilience and enhance their self-efficacy. The second source of self-efficacy evidenced by Bandura [58,59] is social modelling, also referred to as vicarious experiences [58,59]. The author believes that “*seeing people similar to oneself succeed by perseverant effort raises observers’ aspirations and beliefs in their own capabilities*” [58]. In our study, the significance of modelling for inspiring self-belief is emphasised by participants themselves, who highly appreciated the possibility of observing other students and noticed that it created additional occasions to learn and notice their own mistakes. Forbes [60], referring to Bandura’s four sources of information that contribute to an individual self-efficacy, draws attention to the benefits of vicarious experiences in health professional education and observes that social modelling can result in the enhancement of learning efficiency and boosting of learning satisfaction. The third efficacy source is verbal persuasion, which seems relatively easy to use and widely available [59]. Individual feedback after each scenario provoked verbal encouragement from other students, SPs and teachers, helping students overcome self-doubt and focus on the effective outcomes of their performances as doctors. Numerous studies have shown the influence of feedback on learners’ self-efficacy. Kim and Lee [61], for example, stress the effects of positive feedback on nursing students’ positive emotions, increased motivation and higher self-efficacy and call for inclusion, in a balanced way, compliments and positive encouragement in medical training to achieve better emotional and educational goals [61]. Finally, the fourth factor that can affect perceived self-efficacy, referred to by Bandura [59] as emotional arousal, relates to an individual’s emotional and physiological state. Students’ well-being and emotional responses to a given situation, including mood and stress level, determine how they feel about their capabilities to cope with a challenging situation. The fact that students viewed their state of affective arousal as a facilitator of their performance may be explained by Bandura, who states that *“anxiety arousal to threats is likewise diminished by modelling and is even more thoroughly eliminated by experienced mastery achieved through participant modelling”* [59]. Finally, as indicated by Kavanagh and Bower [62], perceived efficacy changes under different moods, and people in positive mood are more likely to improve their belief in capabilities. Moreover, they are bound to accelerate their learning processes [62]. In view of this, we believe the atmosphere of our course, which participants described as supportive, inspiring and convivial, might have played a role in the mood-boosting process and, consequently, increased learners’ self-efficacy.

Next, participants positively evaluated the individual aspects of the course and the effectiveness of the intervention. They believed the class allowed them to improve their cross-cultural competence, which they expect to find useful in their future medical career. During simulations, they felt motivated to help their patients and experienced the realism of the enactments, which was achieved mainly through an outstanding performance delivered by SPs. The feedback, which, as remarked by Jones et al. [19], is the most important section of a simulated session, was a highly rated aspect of the intervention and the comments expressed by SPs were considered the most valuable for students. Pascucci et al. [25], emphasising actors’ contribution to debriefing, relate to the shared experience between students and SPs during a simulated encounter as the main reason for students’ high acceptance and appreciation of feedback delivered by actors. Moreover, as feedback from SPs tends to reflect their personal opinions, stemming from their emotions and observations rather than medical knowledge, it seems less intimidating and more meaningful to students [25].

Our findings also reveal participants’ awareness of the role that the lack of culturally sensitive medical care plays in healthcare and health disparities and call for the intensification of training enhancing cross-cultural knowledge and skills. In fact, in the students’ course improvement suggestions, participants mainly recommend incorporating the class into the obligatory part of the medical curriculum and increasing the number of scenarios during each session. Another interesting finding of our study is that many students see the culture-content simulations as an opportunity to practice and improve their English skills by immersing in authentic communications with English-speaking actors, in which active and attentive listening and appropriate verbal responses become key factors of successful communication. Previous research in applied linguistics has investigated the role of meaningful interactions in foreign language acquisition and the significance of mutual scaffolding in a face-to-face collaborative setting to enhance a language learner’s progress [63–65].

Finally, some differences in participants’ responses were also recognized regarding demographic factors. Gender difference analysis of our findings, for example, pointed out a few diversities in some sections of the questionnaires, with the majority of dissimilarities appearing after the simulations. It also seemed to highlight higher records for female students. In fact, male participants scored higher than their female friends only in their self-assessment of English language skills and abilities to manage patients’ refusal of their own treatment and the treatment of their child that is based on religious grounds. The literature review reveals research indicating that gender can play a significant role in the development and expression of cross-cultural competence, but the suggestions regarding the primacy of either sex remain inconclusive as some studies indicate that women report higher intercultural sensitivity than men [51,66], others contradict this opinion [52]. Next, with regard to students’ age, it was positively correlated with the results in some of the domains of the ISS, which seems consistent with the findings of a previous study [67]. Interestingly, only one positive correlation in terms of participants’ age was noticed in the after-intervention self-efficacy level and it involved students’ knowledge and understanding of cultural, ethnic and national variables. Most differences, including knowledge and understanding of communication methods for building a trusting relationship with patients and their family, knowledge and understanding of the importance of verbal and non-verbal communication, identifying subjective socio-cultural needs of patients, building atmosphere of trust and managing patients’ refusal of their child’s treatment due to their beliefs, were observed before the simulations. Age was also positively correlated with students’ assessments of the course aspects, such as course usefulness, scenarios, the possibility of observing other students and feedback from them. The last finding seems particularly interesting in view of the fact that the majority of students indicated feedback from students as less appreciated than comments received from SPs and teachers. Finally, the study year did not influence participants’ ISS score in our research. By contrast, Pineda et al. [68], in their analysis conducted on the students of three health professions, notice that they tend to exhibit lower ISS scores in the final year of their study compared to the first year, while Aktaş et al. [53] present contradictory findings, showing an increasing level of cross-cultural sensitivity as students approach graduation. Respondents’ year of study positively correlated, however, with their self-efficacy ratings, where, similarly with age, most of them were seen before the interventions and involved knowledge and understanding of communication methods for building a trusting relationship with patients and their family, knowledge and understanding of the importance of verbal and non-verbal communication, identifying subjective socio-cultural needs of patients, building atmosphere of trust, managing patients’ refusal of their child’s treatment due to their beliefs and talking to patients and their family with the use of active listening techniques. After the course, we noticed a correlation between the study year and students’ self-assessment of their abilities to talk to patients attached to natural treatment methods and traditional health beliefs. Then, study year positively correlated with participants’ level of agreement with three statements describing the course; firstly, they had a chance to notice their own mistakes by watching other students; secondly, they felt motivated to do their best to help patients; thirdly, scenarios were not demanding enough and did not constitute a challenge for them. This may have been the consequence of more formative opportunities that students have been offered through their education.

From the findings of this study, the following implications and suggestions may be put forward for decision-makers and future authors. Firstly, the students’ willingness to participate in the class, their active engagement and subsequent favorable feedback highlighted their awareness of the importance of acquiring cross-cultural knowledge, particularly in response to Poland’s demographic trends. These findings should prompt policy-makers to make efforts to increase the frequency of cross-cultural communication training in the medical curriculum to address students’ needs. Then, the methods of instruction employed to enhance skills for effective communication in diverse cultural contexts should be evaluated and the involvement of simulated sessions should be increased as they constitute a valuable and effective teaching approach during cultural training. This should also draw more attention of academic instructors to the significance of comprehensive training offered to simulated patients on role presentation, including culturally contextualized portrayals, and delivering insightful feedback. Next, students’ appreciation of the opportunity to communicate in English in an authentic setting with real patients should be taken into account. English language instruction at our and other universities could be improved by immersing learners into realistic simulations with trained patients portraying real-life clinical scenarios. Finally, although the findings of our study indicate a considerable increase of the self-efficacy of cultural competencies, further research should focus on other factors that may influence students’ perceptions, including various sociodemographic factors as well as English language proficiency. Verification whether similar growth of the self-efficacy of cross-cultural competence following sessions with SPs would also be present among medical students of other Polish universities would also seem worthwhile.

### Limitations

This study has some limitations that should be acknowledged. Firstly, although the response rate was very high (98.6%), the sample size was small, which resulted from the medical curriculum constraints and medical students’ overall busy schedules, which might have prevented some of them from enrolling in the course. However, the considerable interest in our study module and the scarcity of similar courses offered to medical students may indicate the need for curricular modifications. Furthermore, the number of male respondents was lower than that of females, which, in fact, corresponds with the general demographic characteristics of the medical student population at our university. Then, the vast majority of participants were in the second year of their study, leaving a small group of respondents in higher study years, which was the result of substantial enthusiasm and interest in the course among younger students who had so far limited experience with simulations due to the early stages of education they were at. Next, our project relied only on students’ self-report.

Moreover, our study revealed that cross-cultural competence differed according to gender, age and study year of our respondents. The analysis is bivariate, however, and cannot eliminate any potential confounding between those variables. Furthermore, we did not account for any other sociodemographic aspects that might also have affected the competence. Therefore, further studies are needed to evaluate the significance of such factors as students’ upbringing, high school and primary school education, past personal experience, including foreign country experience like travelling abroad, living abroad, going abroad through education, etc. Also, it would be desirable to determine whether and how English language proficiency influences interaction confidence and affects the level of cross-cultural skills. Another concern is that participants were all students of only one medical university, and consequently, the conclusions drawn from our findings may not be representative of all Polish medical students. Therefore, further studies are needed to extend our analysis to a broader population.

## Conclusions

This study demonstrates that students’ intercultural sensitivity, awareness and self-efficacy in terms of cross-cultural knowledge and skills can be positively affected by experiences offered by simulation-based education interventions. Although more research is needed to assess the effectiveness of culture competence courses and particular predictors of cross-cultural competence among medical students, our study shows that interventions with SPs are not only highly appreciated among learners but also help to support their confidence in communicating with patients from diverse cultures. Since the quality of health service could be negatively affected by unsuitable attitudes and actions of unprepared physicians, the need for the inclusion of cross-cultural competence content in the medical curriculum should be stressed.
